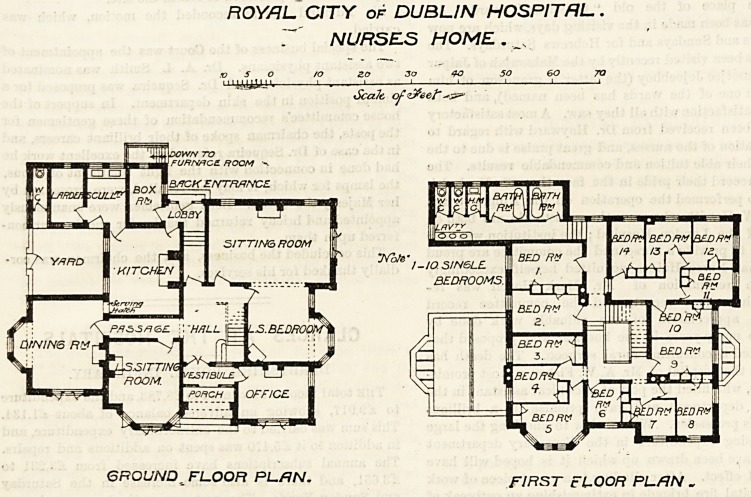# Nurses' Home at the City of Dublin Hospital

**Published:** 1902-09-13

**Authors:** 


### The Institutional Workshop.

### NURSES' HOME AT THE CITY OF DUBLIN HOSPITAL.

The ground floor area of this block is not much more than 64 feet by 50 feet, and the first and second floors are less because one of the rooms is not carried up. It is one of the most compact buildings we ever examined, and contains more accommodation for nurses than, probably, any other home of like dimensions. Every square foot has been utilised. There is a good, wide porch having on its right the offices, and on its left the lady superintendent's sitting-room. Then a passage leading to a dining-room about 20 feet by 18 feet. Next to that is the kitchen with a serving hatch to dining-room. Beyond are box-room, scullery, larder, and servants' closet. The sitting-room, about 25 feet by 18 feet, occupies the opposite angle. The hall and staircase are near the centre of the building and are apparently lighted from the roof.

GROUND FLOOR PLAN. FIRST FLOOR PLAN.

The first floor contains 14 bedrooms, 2 bath-rooms, sink, closets and lavatory. The closets at all events, if not the bath-rooms, ought to have been separated from the rest of the building by a ventilated passage. The second floor is presumably the same as the first floor. There will therefore be a total of 28 bedrooms. Some few of these will be for the domestic staff, but assuming that 24 nurses are in residence, we venture to doubt whether either the dining-room or sitting-room is large enough, even if we bear in mind that the whole of the nurses are rarely in either of these rooms at the same time. Naturally, from what we have said of the ground space occupied, it is to be expected that some of the nurses' rooms look a trifle small; but there is, perhaps, no nurse who would not rather have a small room to herself than share one three times as large with another nurse.

The home is built in the recreation grounds of the hospital, and the foundation-stone was laid last year by the Princess Christian. The elevations are of red brick relieved by buff terra-cotta.

The rooms are warmed by hot-water radiators, and lighted by the electric light.

The architect is Mr. Albert E. Murray, of Dublin. Neither the contractor's name nor the cost of the building is stated.

## Figures and Tables

**Figure f1:**